# Performance Evaluation of a Bioinspired Geomagnetic Sensor and Its Application for Geomagnetic Navigation in Simulated Environment

**DOI:** 10.3390/s24196477

**Published:** 2024-10-08

**Authors:** Hongkai Shi, Ruiqi Tang, Qingmeng Wang, Tao Song

**Affiliations:** 1 Beijing Key Laboratory of Bioelectromagnetism, Institute of Electrical Engineering, Chinese Academy of Sciences, Haidian District, Beijing 100190, China; 2 University of Chinese Academy of Sciences, Shijingshan District, Beijing 100049, China

**Keywords:** bioinspired sensor, geomagnetic navigation, finite element, simulated navigation platform

## Abstract

For geomagnetic navigation technology, taking inspiration from nature and leveraging the principle of animals’ utilization of the geomagnetic field for long-distance navigation, and employing biomimetic technology to develop higher-precision geomagnetic sensors and more advanced navigation strategies, has emerged as a new trend. Based on the two widely acknowledged biological magnetic induction mechanisms, we have designed a bioinspired weak magnetic vector (BWMV) sensor and integrated it with neural networks to achieve geomagnetic matching navigation. In this paper, we assess the performance of the BWMV sensor through finite element model simulation. The result validates its high measurement accuracy and outstanding adaptability to installation errors with the assistance of specially trained neural networks. Furthermore, we have enhanced the bioinspired geomagnetic navigation algorithm and proposed a more advanced search strategy to adapt to navigation under the condition of no prior geomagnetic map. A simulated geomagnetic navigation platform was constructed based on the finite element model to simulate the navigation of the BWMV sensor in geomagnetic environments. The simulated navigation experiment verified that the proposed search strategy applied to the BWMV sensor can achieve high-precision navigation. This study proposes a novel approach for the research of bioinspired geomagnetic navigation technology, which holds great development prospects.

## 1. Introduction

In the field of geomagnetic navigation technology, the accurate measurement of geomagnetic fields serves as the foundation for achieving autonomous navigation in geomagnetic environments [1,2]. The ongoing advancement of fundamental physics research and modern electronic technology is driving the continuous development of magnetic field detection technology, enhancing geomagnetic measurements towards higher precision [3,4]. Simultaneously, concomitant with the advancement of biomimetic technology, research on obtaining inspiration from biological systems and applying them to geomagnetic navigation is constantly evolving, including the development of bioinspired geomagnetic sensors [5,6]. Bioinspired geomagnetic sensors draw inspiration from the magnetic sensing mechanism of animals and offer a novel approach to enhancing the performance of geomagnetic sensors by imitating the utilization of geomagnetic field in animal navigation process.

The two main magnetic sensing mechanisms currently include the magnetic particle mechanism and the free radical pairs mechanism. The magnetic particle mechanism refers to the fact that magnetic particles in animal bodies trigger neural signals under external magnetic fields, thus achieving perception of the magnetic field [7,8]. The free radical pair mechanism refers to the magnetic sensing mechanism based on chemical magnetic receptors. This mechanism relies on the free radicals induced by photoreceptors to form intermediates [9]. The earliest discovered intermediate of free radicals is cryptochrome (Cry), which has been increasingly demonstrated to be involved in the mechanism by which migratory songbirds sense the direction of the geomagnetic field [10,11].

A further crucial area of investigation within the field of bioinspired geomagnetic navigation is the analysis and emulation of navigation strategies employed by living organisms. Inspired by animals such as pigeons and turtles to utilize geomagnetic information for long-distance migration and homing, current research is increasingly focused on achieving navigation without prior geomagnetic maps. In this context, the pivotal challenge pertains to the effective utilization of magnetic field data in the process of path planning and correction. In recent years, researchers have studied various algorithms to address these issues [12,13,14], including the probability evolution strategy [15], the model predictive control algorithm [16], and the differential evolution algorithm [17].

In the study of the magnetic sensing mechanism in birds, our research team found that there may be some coupling between the magnetic particle mechanism and the free radical mechanism in the magnetic sensing process of birds [18]. Based on the coupling magnetic sensing mechanism, we designed a bioinspired weak magnetic vector (BWMV) sensor which consists of a magnetic rod and a sensor plane, simulating two different magnetic sensing mechanisms. Geomagnetic navigation experiments were conducted under a simulated geomagnetic navigation device based on three-dimensional Helmholtz coils, verifying the effectiveness of the sensor in geomagnetic matching navigation [19]. For the BWMV sensor, further studies are required regarding its robustness and adaptability to installation errors. Additionally, researching bioinspired geomagnetic navigation strategies suitable for this sensor to achieve geomagnetic navigation without prior geomagnetic maps is also a direction worthy of exploration.

As mathematical modeling and simulation serve as complementary means for investigating geomagnetic navigation [20], the finite element simulation was applied to simulate the measurement of the sensor in a magnetic field environment. Additionally, neural networks were used to convert the values measured directly by the BWMV sensor into the measurement results of external magnetic field parameters. By comparing the measurement results of the BWMV sensor under different noise environments, the robustness of the sensor was studied, and the impact of installation errors on the sensor was discussed.

On this basis, we propose a navigation algorithm without prior geomagnetic maps and construct a simulated geomagnetic navigation platform based on a finite element simulation model to verify the geomagnetic navigation performance of the algorithm applied to the BWMV sensor. The results demonstrate that the BWMV sensor with high-precision magnetic measurement performance combined with this navigation algorithm has excellent adaptability in simulating geomagnetic environments and can accurately complete navigation according to the set accuracy.

## 2. The Design and Simulation of the BWMV Sensor

### 2.1. The Basic Principles of the BWMV Sensor

**Figure 1:**
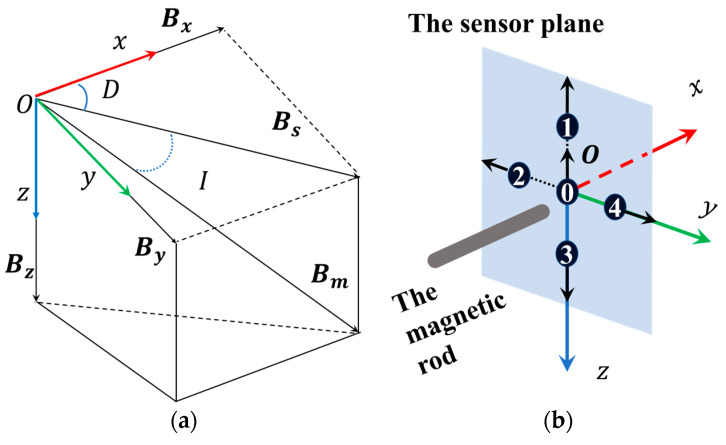

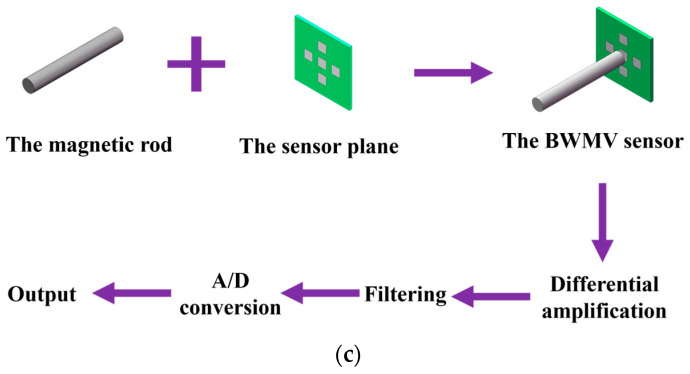
(**a**) Schematic diagram of the geomagnetic field vector and elements; (**b**) structure diagram of the BMWV sensor; (**c**) principle of the BMWV sensor.

To discuss the BWMV sensor, it is necessary to define the basic concepts of the geomagnetic field. For any position on the Earth, there exists a geomagnetic field vector, which can be defined as $B_{m}$, as shown in Figure 1a. We establish a Cartesian coordinate system $\Sigma_{Oxyz}$ that coincides with the geographic coordinate system to analyze the geomagnetic field vector, where the positive directions of the x-axis, y-axis, and z-axis correspond to geographic north, geographic east, and perpendicular downward, respectively. Therefore, $B_{x}$, $B_{y}$, and $B_{z}$ are the projections of $B_{m}$ on the three coordinate axes, respectively. In addition, the projection of $B_{m}$ on the horizontal plane is defined as $B_{s}$, and the mutual relationship between geomagnetic components can also be represented by angles. The angle between $B_{s}$ and $B_{x}$ is defined as the magnetic declination D, which is positive in a clockwise direction. The angle between $B_{m}$ and $B_{s}$ is defined as the magnetic inclination I, which is positive when oriented downward along the horizontal plane.

In accordance with the established definition of a geomagnetic field, complete geomagnetic information can typically be represented by two sets of scalars, $B_{m}$, D, I, and $B_{x}$, $B_{y}$, $B_{z}$. The relationship between these variables can be expressed as follows:

(1) $$\left\{\begin{array}{c} B_{x}=B_{m}\cos I\cos D\\ B_{y}=B_{m}\cos I\sin D\\ B_{z}=B_{m}\sin I. \end{array}\right.$$

In the actual process of measuring the geomagnetic field, the geomagnetic field measured by the sensor is related to the attitude of the sensor. This involves the conversion and calculation of the coordinate system between the sensor and the geomagnetic field. In this paper, to simplify the problem, the two coordinate systems are set to be consistent.

The structure and principle of the BWMV sensor are illustrated in Figure 1b and Figure 1c, respectively. The BMWV sensor is composed of two principal elements: a magnetic rod and a sensor plane. The magnetic rod emulates the mechanism of magnetic particles, changing the distribution of the surrounding magnetic field in a magnetic field environment. This enables the detection of magnetic field information in a plane. The sensor plane emulates the mechanism of free radical pairs. In this mechanism, the proportion and distribution of singlet and triplet states of free radical pair products are influenced by the external magnetic field angles. Therefore, by detecting the distribution of magnetic field on the plane under the influence of the magnetic rod, the perception of magnetic field information can be realized. The combined action of the magnetic rod and sensor plane allows the BWMV sensor to simulate the joint magnetic sensing mechanism of magnetic particles and free radicals. The signal generated by the sensor under a magnetic field is subjected to differential amplification, filtering, A/D conversion, and finally output.

The finite element method is a numerical analysis method for solving partial differential equations and simulating physical systems. The method discretizes the continuous solution domain into finite elements with simple shapes such as triangles or quadrilaterals. By approximating unknown functions on each element, it generates element equations and combines them into an algebraic equation system, obtaining the approximate solutions.

**Figure 2:**
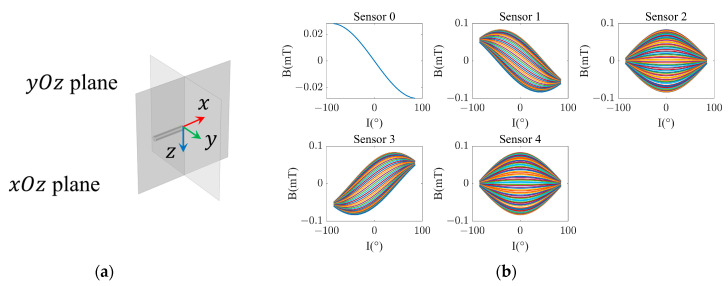
(**a**) The relative position between the magnetic rod and the planes; (**b**) the simulated measured values of sensors with fixed magnetic flux density.

In this paper, a general simulation software based on advanced numerical methods (COMSOL Multiphysics, COMSOL Inc., Stockholm, Sweden) is employed as a finite element tool for the investigation of the BWMV sensor. As illustrated in Figure 2a, a small cylinder simulates a magnetic rod in COMSOL Multiphysics, which is in a cube measuring 500 × 500 × 500 mm. The center of the cube coincides with the coordinate origin, and the coordinate axis passes through the center of each face. The cube simulates the air around the magnetic rod, and its boundary conditions are set to external magnetic flux density. The sensor plane is located on the yOz plane, and the distance between the end of the cylinder and the yOz plane is set to 1.5 mm. The relative magnetic permeability of the cylinder is 2000 with a radius of 2.5 mm and a length of 30 mm.

With a fixed total magnetic flux density of $B_{m}$ = 0.05 mT, parameterized scans were performed on the finite element model by changing the magnetic declination D and magnetic inclination I, where D = (−180, 180°), I = (−90, 90°), and the step sizes are 5°. The results are shown in Figure 2b, in which each curve represents the variation in the magnetic flux density of the sensor with the magnetic inclination I while maintaining the magnetic declination D constant. For sensor 0, all curves are identical, indicating that the measured value of the sensor is independent of D. However, the simulated measured values of sensors 1 to 4 vary with D and I, indicating that the simulated magnetic field measurements at these points can effectively reflect the changes in the magnetic field angle.

### 2.2. The Accuracy and Robustness of the BWMV Sensor

As the directly measured values of the BWMV sensor are the magnetic flux densities of five sensors, it is still necessary to convert these directly measured values of the sensors into the measurement results of the corresponding magnetic field parameters $B_{m}$, D, and I. The artificial neural networks are used as a tool to implement this process. Artificial neural networks, as a mathematical model derived from the cognitive functions of living organisms, have a wide range of applications in fields such as signal and image processing due to their powerful data analysis capabilities [21,22]. With the emergence of big data and artificial intelligence, neural networks have been widely utilized in geomagnetic navigation technology due to their universality in solving practical problems and good intelligent characteristics [23,24].

In this paper, we continue to employ the three-layer BP neural network that was utilized in our previous work [19]. In a three-layer neural network, the number of input nodes and output nodes must be determined based on the specific problems and the task requirements. For the input layer, the number of nodes needs to be set in a way that fully expresses the feature information of the input data. As for the output layer, the number of nodes should be configured so that the output results are easy to understand and interpret, providing valuable information for decision making. The number of nodes in the hidden layer, which directly affects the accuracy of neural networks, needs to be set according to the training data and the actual situation of the neural network [25,26,27]. In this paper, we compare the performance of corresponding validation sets for different numbers of hidden layer nodes to select the most appropriate number of nodes.

When using neural networks to calculate magnetic field parameters, it is considered that the relationship between the magnetic inclination angle and the magnetic induction intensity in a single direction may include an arctangent function, which increases the scale of the artificial neural network and reduces the calculation accuracy. Accordingly, the magnetic field components in three directions $B_{x}$, $B_{y}$ and $B_{z}$ are employed as output values for calculation, and subsequently converted to obtain $B_{m}$, D, and I. According to Equation (1), the conversion formula can be expressed as follows:

(2) $$B_{m}=\sqrt{{B_{x}^{2}+B_{y}^{2}+B}_{z}^{2}},$$

(3) $$D=\left\{\begin{array}{cc} \arctan\frac{B_{y}}{B_{x}}+\pi & \left(B_{x}<0\right)\\ -\frac{\pi }{2} & \left(B_{x}=0,B_{y}<0\right)\\ \frac{\pi }{2} & \left(B_{x}=0,B_{y}>0\right)\\ \arctan\frac{B_{y}}{B_{x}} & \left(B_{x}>0\right) \end{array}\right.,$$

(4) $$I=arcsin\left(\frac{B_{z}}{B_{m}}\right).$$

The objective of the neural network is to convert the magnetic flux densities of five points into corresponding magnetic field parameters; that is, the nodes of the input layer represent the magnetic flux densities of the five points, and the nodes of the output layer correspond to the magnetic field parameters $B_{x}$, $B_{y}$ and $B_{z}$. Therefore, the number of nodes in the input layer and output layer is 5 and 3, respectively.

Furthermore, the root mean squared error (RMSE) is employed to evaluate the accuracy of the neural network model in calculating the geomagnetic parameters measured by the sensor. The calculation formula for RMSE is as follows:

(5) $$RMSE=\sqrt{\frac{1}{l}\sum_{i=1}^{l}{\left(y_{i1}-y_{i2}\right)}^{2}},$$

where l is the data length, and $y_{i1}$ and $y_{i2}$ represent the i-th corresponding values of the two datasets, which represent the set magnetic field parameters and the calculated magnetic field parameters, respectively.

The input values and corresponding output values are randomly partitioned in a ratio of 0.7:0.15:0.15 for the training set, validation set, and test set with respective quantities of 1739, 373, and 373, respectively. The training process of neural networks involves the initialization of the neural network and the application of learning algorithms to train ordinary parameters on the training set with the objective of minimizing the model’s error on the training set. Subsequently, the generalization ability of the network must be validated using the validation set, and the hyperparameters can be adjusted based on model performance. These two steps should be repeated until the network achieves a lower generalization error on the validation set, at which point the entire training process will have concluded. After adjusting various hyperparameters such as transfer functions and the number of hidden layer nodes to train the neural network, the network is evaluated using the test set, and the network with the smallest RMSEs is selected. The number of nodes in the hidden layer is 20. The transfer function between the input layer and the hidden layer is “purelin”, and the transfer function between the hidden layer and the output layer is “satlins”. The RMSEs of $B_{m}$, D, and I were 4.11 × 10^−5^ μT, 0.10″ and 0.23″, respectively.

The robustness of the current scheme was studied using Gaussian white noise. Before calculating the magnetic field parameters, different proportions of Gaussian white noise were added to the magnetic fields at five locations. The RMSEs between the set and calculated values of the magnetic field parameters after adding noise are presented in Table 1 for comparison. The inversion accuracy of magnetic induction intensity is high with low external magnetic field noise, while the error also significantly increases as the noise power increases.

### 2.3. The Adaptability of the BWMV Sensor to Installation Errors

It is possible that there may be certain deviations in the relative positions and angles of each sensor on the plane, which could also affect the accuracy of inversion. In the case of positional errors, a deviation of 1% of δ (0.04 mm) is assumed. For angular errors, a rotation angle β of ±1° is considered with a positive value indicating a clockwise direction. The research method is to deviate the position or angle of a single point at a time while maintaining the ideal positions of the remaining points. The RMSE of the magnetic field parameters is then calculated in comparison compared to the set values.

Firstly, the impact of installation errors of the center position sensor, i.e., sensor 0, on the calculation results is discussed. The calculation results of magnetic field parameters caused by different errors are presented in Table 2. The offset of sensor 0 on the y-axis has the least impact on the RMSEs of the magnetic field parameters. However, the deviation of RMSEs caused by the offset on the x-axis and the rotation of the angle is relatively large, indicating that the neural network trained using magnetic field parameters at ideal positions is susceptible to the installation errors of sensor 0.

**Figure 3:**
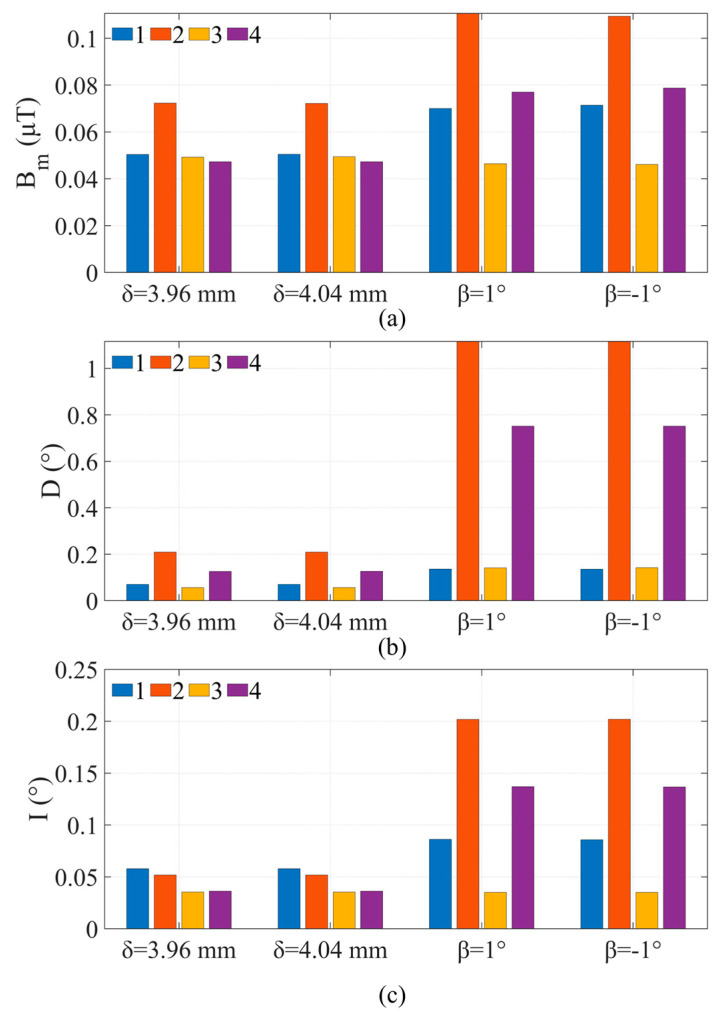
RMSEs of (**a**) $B_{m}$, (**b**) D and (**c**) I affected by non-ideal relative position of sensors.

Regarding the remaining four sensors, the research method entails alternating the movement of a single point while maintaining the optimal positioning of the remaining points. The result is illustrated in Figure 3. The influence of installation errors on the total magnetic induction intensity $B_{m}$ is relatively minimal. For the magnetic declination D, the RMSEs resulting from installation errors of sensors 1 and 3 are within 0.2°, while the impact of angular rotation of sensors 2 and 4 is significant. In the case of β = ±1°, the RMSE can exceed 1°, which is far higher than the impact of sensors 1 and 3 under the same conditions. For magnetic inclination I, the RMSEs caused by sensor installation errors are within 0.25°. In addition, due to the larger RMSEs of the magnetic field parameters caused by the position and angle errors of sensor 2 compared to other sensors, it is speculated that there may be a higher correlation weight for sensor 2 in the neural network, which makes the inversion results of sensor 2 more sensitive to its errors.

Due to the high flexibility and universality of neural networks in computing data, the installation errors can be addressed by retraining the actual measured values to obtain a new network and apply it, which is like making a correction based on installation errors. To verify its feasibility, we simulated the directly measured values of the BWMV sensor corresponding to the installation errors by simulation and used them to train new neural networks. For the 22 sets of data with installation errors, the neural networks were retrained by adopting the same method as that used for the data of the ideal position. The new neural networks were utilized to invert the data with installation errors to obtain the RMSEs. Table 3 shows the maximum values of RMSE of $B_{m}$, D, and I corresponding to different installation errors of each sensor, which are less than 0.02 nT, 0.2″ and 0.1″, respectively. It indicates that for the BMWV sensor with possible installation errors, the accurate conversion of magnetic field parameters from sensor measurements can also be accomplished by training a suitable neural network.

## 3. The Simulated Geomagnetic Navigation Platform and Bioinspired Navigation Algorithms

### 3.1. The Simulated Geomagnetic Navigation Platform

**Figure 4:**
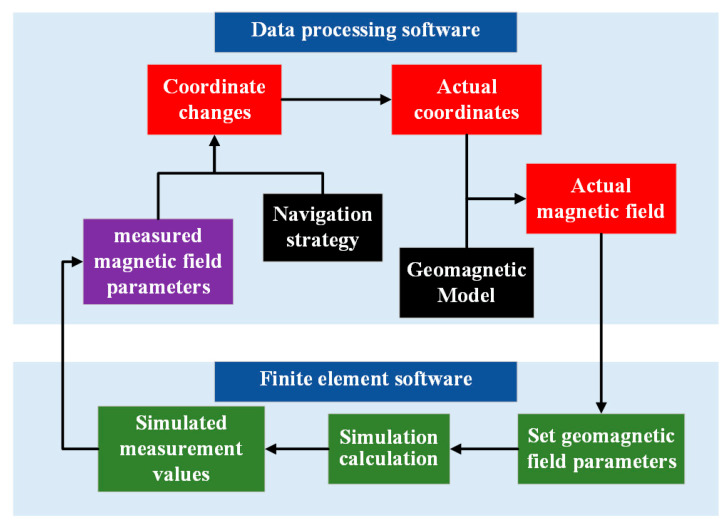
Schematic diagram of the simulated geomagnetic navigation platform.

The BWMV sensor was tested and validated using finite element models, and a neural network model was trained to calculate geomagnetic field parameters, demonstrating that the BWMV sensor can achieve high-precision geomagnetic measurements. To further verify the navigation performance of the BWMV sensor, we constructed a simulated geomagnetic navigation platform based on finite element models and jointly programmed it with data processing software. The principle of the platform is illustrated in Figure 4.

The simulated geomagnetic navigation platform is comprised of four subsystems, including geomagnetic field models, the carrier model, the sensor model, and navigation algorithms. Among them, the sensor model is simulated by the finite element method to simulate the detection results of the sensor, which is implemented by the finite element software, and the rest is completed through data processing software. The geomagnetic field model adopts the International Geomagnetic Reference Field (IGRF) [28]. The carrier model is utilized to define the motion characteristics of the carrier where the sensor is located as well as to simulate the actual motion of the carrier in the simulation. These three models are integrated with navigation algorithms to jointly simulate the operation of navigation.

The simulation platform is based on the Windows 10 operating system with a CPU clock frequency parameter of 2.50 GHz and 16 GB of memory. For the platform, the input parameters comprise the coordinates of the starting point and the target point, the navigation algorithm. The output parameters consist of the measurement values, actual coordinates, as well as the judgment results and motion strategies related to the navigation algorithm.

In the simulation navigation process, the sensor performs simulated “movement”, and the data processing software calculates new actual coordinates based on the changes in coordinates. The geomagnetic model is then employed to calculate the actual magnetic field corresponding to the coordinate point. The calculated geomagnetic field parameters can be set as the background magnetic field of the sensor simulation model. Through simulation by finite element software, the simulated measured values of the sensors can be obtained. Based on these simulated measured values, the measurement results of the magnetic field parameters at the current position are calculated, and it is determined whether the destination has been reached. If the requirements are met, the navigation ends; otherwise, the navigation continues.

The simulated geomagnetic navigation platform offers the advantage of enabling the navigation performance of sensors and navigation algorithms to be simulated under ideal conditions. Furthermore, the robustness of the algorithm can be verified by introducing interference. The entire experiment is conducted through a computer and can simulate navigation experiments in any environment.

### 3.2. The Basic Process of Bioinspired Geomagnetic Navigation

The traditional method of geomagnetic navigation is conducted with a geomagnetic map that is known to be accurate. From the perspective of mimicking biological navigation, we studied the geomagnetic navigation strategy in the absence of prior geomagnetic maps and verified it with the BWMV sensor on the simulated geomagnetic navigation platform, thus preparing for the next step of geomagnetic navigation in laboratory and even practical environments.

Before discussing the navigation strategies, it is necessary to clarify that there are two distinct viewpoints in the simulation of navigation, namely the perspective of the platform and that of the sensor. The former is privy to all the parameters during the navigation process, while the latter is shrouded in uncertainty and can only judge its own state through the measured values.

The basic process of simulated navigation is as follows:

1. Determine the coordinates of the starting and target points, the target accuracy and initial navigation spacing of the navigation, and the corresponding navigation strategy;
2. Set the latitude and longitude of the starting and target points and the geomagnetic model applied to calculate the geomagnetic field parameters of the actual position during navigation;
3. Set the direction of movement according to the preset navigation strategy and adjust the navigation spacing according to the actual situation;
4. The sensor performs simulated movement based on the set heading and navigation spacing, while the simulated geomagnetic navigation platform calculates the actual position and corresponding latitude and longitude after movement;
5. The geomagnetic field parameters of the new location are calculated by the geomagnetic model and set as the background magnetic field of the sensor model;
6. Simulate the sensor model and convert the simulated measured values of the sensors into the simulated measurement results of the magnetic field parameters.

Calculate the deviation between the simulated measurement results of the magnetic field parameters and the magnetic field parameters of the target point and determine whether the set target accuracy has been achieved. If it has been achieved, it is considered that the target point has been reached and the navigation ends. Otherwise, repeat steps 3 to 6 to continue the navigation.

## 4. Navigation Strategy and Simulated Experiment Verification

### 4.1. The Search Strategy in Geomagnetic Navigation without Prior Geomagnetic Map

When discussing the accuracy of navigation, it is necessary to consider the distance between different positions. In the case of a relatively smaller area, it can be treated as a plane to simplify the problem. The distance corresponding to every 1° change in latitude is 111 km, and the distance corresponding to every 1° change in longitude is 111 cos φ kilometers, where φ is the latitude. Therefore, given the latitude and longitude of two points, the distance between them can be calculated.

$B_{m}$, D and I are used together as the basis for determining whether the endpoint is approaching. The specific method determines the search direction by calculating the absolute differences between the three geomagnetic parameters of the sensor position and the target point during navigation. Define n as the sequence number during the navigation process. $P_{n}$ represents the position of the sensor during the n-th navigation, and Q represents the target point. The measurement results (total magnetic flux density, magnetic declination, and magnetic inclination) of the sensor at point $P_{n}$ are denoted by $B_{mn}$, $D_{n}$, and $I_{n}$, respectively, and the geomagnetic parameters at the target point Q are denoted by $B_{mQ}$, $D_{Q}$, and $I_{Q}$, respectively. The absolute differences $\Delta B_{mn}$, $\Delta D_{n}$, and $\Delta I_{n}$ are defined as follows:

(6) $$\left\{\begin{array}{c} \Delta B_{mn}=\left|B_{mn}-B_{mq}\right|\\ \Delta D_{n}=\left|D_{n}-D_{q}\right|\\ \Delta I_{n}=\left|I_{n}-I_{q}\right| \end{array}\right..$$

At the starting point, n is initialized as 0. The absolute differences between the geomagnetic parameters at the starting point and the target point are denoted as $\Delta B_{m0}$, $\Delta D_{0}$, and $\Delta I_{0}$, respectively. The variable $S_{n}$, which represents the degree of proximity of magnetic field parameters, is defined as follows:

(7) $$S_{n}=\max\left(\frac{\Delta B_{mn}}{\Delta B_{m0}},\frac{\Delta D_{n}}{\Delta D_{0}},\frac{\Delta I_{n}}{\Delta I_{0}}\right).$$

When $S_{n}$ is less than or equal to the set value $S_{0}$, it is considered that the navigation task has been completed. Otherwise, the navigation will continue. During the navigation process, set the carrier where the sensor is located to move in a straight line between two measurements with the angle $\alpha_{n}$, the motion unit vector $v_{n}\;(\cos\alpha_{n},\sin\alpha_{n})$ and the navigation spacing $L_{n}$. The angle $\alpha_{n}$ is introduced to represent the angle between the motion direction of the sensor and the latitude line with counterclockwise being positive. To balance the speed and efficiency of navigation, adjustments will be made to $L_{n}$ based on $S_{n}$, that is

(8) $$L_{n}=\left\{\begin{array}{cc} L_{0} & \left(S_{n-1}>1\right)\\ {(S}_{n-1}/{S_{0})L}_{0} & \left(S_{n-1}\leq 1\right) \end{array}\right..$$

In this paper, the measured values of the sensors are converted into magnetic field parameters. Then, the gradient of the magnetic field parameters is calculated to plan the direction of navigation. The expression formulas for gradients $\nabla B_{mn}$, $\nabla D_{n}$ and $\nabla I_{n}$ are as follows:

(9) $$\left\{\begin{array}{c} \nabla B_{mn}=(\Delta B_{mn}-\Delta B_{m(n-1)})/L_{n}\\ \nabla D_{n}=(\Delta D_{n}-\Delta D_{n-1})/L_{n}\\ \nabla I_{n}=(\Delta I_{n}-\Delta I_{n-1})/L_{n} \end{array}\right..$$

To study the gradient changes of the three magnetic field parameters, the variable $k_{n}$ is defined as follows:

(10) $$k_{n}=sgn\left(\nabla B_{mn}\right)+sgn\left(\nabla D_{n}\right)+sgn\left(\nabla I_{n}\right),$$

where the function sgn(a) is used to calculate the sign of the real number a, which is expressed as

(11) $$sgn(a)\left\{\begin{array}{cc} 1 & a>0\\ 0 & a=0\\ -1 & a<0 \end{array}\right..$$

In an ideal state, as the carrier gradually approaches the target point along the optimal direction, the geomagnetic parameters also become closer to the target point, and the corresponding motion vector is $u_{n}$, with $k_{n}$ = −3. Since navigation is performed without prior geomagnetic maps and the optimal direction cannot be determined, the search strategy continuously adjusts $\alpha_{n}$ to make the moving vector $v_{n}$ approach $u_{n}$ as closely as possible to the optimal direction.

As the accurate direction is uncertain at the beginning of navigation, the initial two steps are executed at random angles, which are designated as $\alpha_{1}$ and $\alpha_{2}$. When n > 2, $\alpha_{n}$ will be adjusted based on the parameters calculated in the previous steps.

When $k_{n}$ = −3, it is considered that the sensor is moving in the correct direction. In this case, set $\alpha_{n+1}=\alpha_{n}$. When $k_{n}$ = 3, it is believed that the direction of motion of the sensor is opposite to the ideal direction. Then, set $\alpha_{n+1}=\alpha_{n}$+ 180°. For the case where $k_{n}$ ≠ ±3, the adjustment plan for angle $\alpha_{n+1}$ is as follows:

If $\alpha_{n}=\alpha_{n-1}$ or $k_{n}$ = $k_{n-1}$, it indicates that the direction at this point is no longer optimal. In such a situation, a random search should be conducted and $\alpha_{n+1}$ can be set as

(12) $$\alpha_{n+1}=(\alpha_{n}+\alpha_{n-1})\;/2+\gamma ,$$

where γ is a random angle, ranging from −90 to 90°.

**Figure 5:**
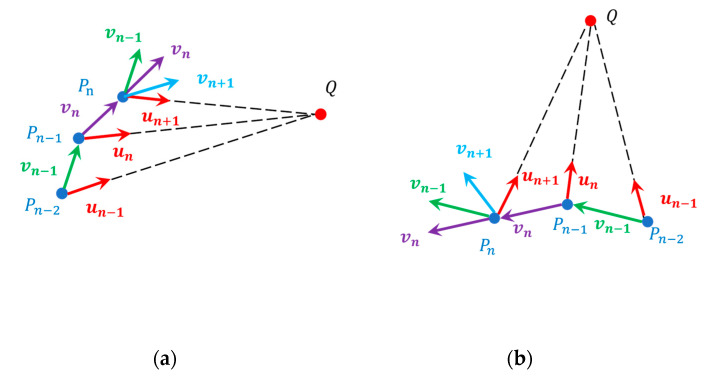
The search strategy when (**a**) $k_{n}$ > $k_{n-1}$ and (**b**) $k_{n}>k_{n-1}$ during navigation.

For the case where $\alpha_{n}\neq \alpha_{n-1}$ and $k_{n}\neq k_{n-1}$, we use Figure 5 to explain the principle of setting $\alpha_{n+1}$. In Figure 5, $P_{n-2}$, $P_{n-1}$, and $P_{n}$ represent three consecutive positions during the motion process, and Q represents the target point. Meanwhile, $u_{n-1}$, $u_{n}$ and $u_{n+1}$ represent the optimal motion vector, and $v_{n-1}$, $v_{n}$ and $v_{n+1}$ represent the actual motion vector.

If $k_{n}<k_{n-1}$, as illustrated in Figure 5a, it implies that the angle $\alpha_{n}$ is closer to the ideal direction compared to $\alpha_{n-1}$ at this moment. Therefore,

(13) $$\alpha_{n+1}=\alpha_{n}+sgn\left(\alpha_{n}-\alpha_{n-1}\;\right)\left|\gamma \right|.$$

If $k_{n}$> $k_{n-1}$, as shown in Figure 5b, it indicates that the angle $\alpha_{n-1}$ is closer to the ideal direction than $\alpha_{n}$ at this point. Thus,

(14) $$\alpha_{n+1}=\alpha_{n-1}+sgn\left(\alpha_{n-1}-\alpha_{n}\;\right)\left|\gamma \right|.$$

The angle adjustment method illustrated in Figure 5 is primarily a combination of direction judgment and random search. By comparing $k_{n}$ and $k_{n-1}$, the method determines which direction of motion is closer to the ideal direction in the first two steps and thus selects a reasonable range for random angle search. Within this range, random angle search can cover a wider search space and help find or approach the global optimal solution. The combination of the two ultimately achieves navigation without prior maps.

### 4.2. Results and Analysis of Simulated Navigation Experiment

To verify the navigation strategy and the navigation performance of sensors, set the starting point (116° E, 40° N) and the target point (119° E, 32° N) with a straight-line distance of approximately 929 km between the two points.

**Figure 6:**
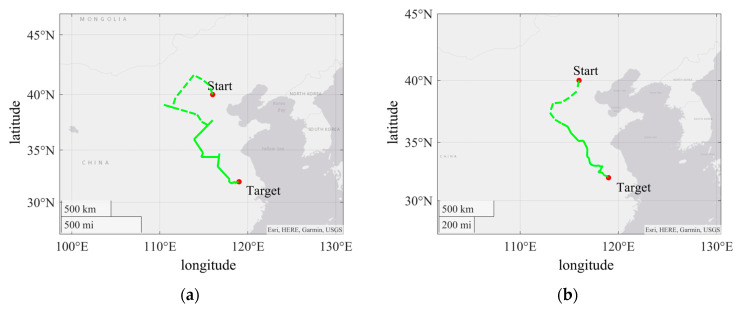
(**a**) The path diagram of simulated navigation under interference free conditions; (**b**) the path diagram of simulated navigation under random angle interference condition.

Set $S_{0}$ = 0.1% and $L_{1}$ = 100 km. The navigation path is shown in Figure 6a. The latitude and longitude of the final point are (118.9947° E, 32.0003° N), and the distance is 0.5009 km, which accounts for approximately 0.054% of the total distance and is close to the predetermined target. This indicates that the BMWV sensor is capable of perform navigation without prior geomagnetic maps.

To verify the robustness of the algorithm, a random angle ω was added as interference during the navigation process, ranging from −30 to 30°. All other conditions remained unchanged throughout the course of the experiment. As shown in Figure 6b, the latitude and longitude of the final point are (118.9939° E, 32.0001° N), and the distance is 0.5753 km, which accounts for approximately 0.061% of the total distance. The existence of random angles has caused certain interference to the accuracy of navigation, but the result also reached the target range, indicating that the algorithm has good robustness against interference in the direction of motion.

The results indicate that this method can accurately reach the destination by comparing the measurement results with the target parameters in the absence of an unknown geomagnetic map combined with a search algorithm. Compared to the case with geomagnetic maps, this method can cope with uncertainty. It can be widely applied to navigation in different geographical environments, ranging from indoor navigation to outdoor exploration. In addition, the application of this method in simulated geomagnetic navigation systems effectively validates the performance of the BWMV sensor in geomagnetic detection and navigation, providing reference for further research and the improvement of sensors.

Moreover, this method has significant advantages in terms of timeliness. During the navigation process, it adjusts the navigation direction and interval based on the measurement results of geomagnetic field parameters to ensure accurate navigation decisions. By adjusting and responding in a timely manner, it can search for suitable navigation paths even in the face of navigation direction errors. This timeliness not only provides critical support for decision making in the navigation process but also greatly improves the reliability and effectiveness of navigation.

## 5. Conclusions

This research primarily hinges on the BWMV sensor based on the joint magnetic induction mechanism, and it further investigates and verifies the performance of the sensor through finite element analysis. A pathfinding algorithm without prior geomagnetic maps is designed and combined with a simulated navigation platform constructed based on finite element software to verify the navigation performance of the sensor in geomagnetic environments, achieving meaningful results.

By employing a finite element model, the BWMV sensor measures and analyzes geomagnetic field parameters via five magnetic field values on the sensor plane. During this process, a neural network is employed to achieve high-precision mapping from the measured values to the magnetic field parameters. The RMSE values of $B_{m}$, D, and I calculated are less than 10^−4^ μT, 0.2″, and 0.3″, respectively, demonstrating extremely high measurement accuracy.

Furthermore, we examined the navigation performance of the BWMV sensor using a simulated geomagnetic navigation platform based on finite element software. The results indicated that the BWMV sensor can conduct high-precision navigation without prior geomagnetic maps. The ratio of the distance between the endpoint and the target point to the starting distance is less than 0.1%. Even with interference from random angles, high accuracy has been achieved. This indicates that the algorithm has good robustness and promising application prospects.

Currently, the BWMV sensor in this paper is merely at the simulation level. Hence, to demonstrate the measurement accuracy and navigation performance of the sensor more effectively is the next step of research. We will upgrade the platform to include discussions on issues such as carrier attitude and sensor error interference to further approach the actual environment. In addition, we will also extend the sensor and navigation experiments to the laboratory and even practical environments, furnishing more valuable experiences for the research of geomagnetic navigation technology.

In summary, this study has made significant contributions to the field of sensor technology. The BWMV sensor has a positive influence on the development of geomagnetic navigation. However, further efforts are needed to fully unlock its potential and facilitate further progress in this crucial research field.

## Figures and Tables

**Table 1:** Comparison of RMSE values for solving geomagnetic parameters using signals with noise.

Signal-to-Noise Ratio	RMSE		
	$B_{m}$ (μT)	D (°)	I (°)
40 dB	0.4038	0.9767	0.4821
30 dB	1.2564	3.5137	1.5509
20 dB	3.6719	11.0137	5.0793
10 dB	9.6884	28.4564	16.1390

**Table 2:** The influence of position error of sensor 0.

Type of Position Error	RMSE		
	$B_{m}$ (μT)	D (°)	I (°)
y = 0, z = −0.04 mm	0.2453	0.1258	0.4803
y = −0.04 mm, z = 0	0.0019	0.0016	0.0016
y = 0, z = 0.04 mm	0.2451	0.1259	0.4806
y = 0.04 mm, z = 0	0.0008	0.0006	0.0014
β = 1°	0.1653	0.0511	0.3166
β = −1°	0.1646	0.0510	0.3168

**Table 3:** Maximum values of RMSEs of data with installation errors: solved by retrained new networks.

Sensor Number for Installation Error	Maximum Value of RMSE		
	$B_{m}$ (nT)	D (″)	I (″)
0	0.0102	0.1298	0.0521
1	0.0170	0.1157	0.0702
2	0.0130	0.1229	0.0468
3	0.0093	0.1549	0.0460
4	0.0065	0.1671	0.0303

## Data Availability

The data presented in this study are available on request from the corresponding author.

## References

[1] Zhao H., Zhang N., Xu L., Lin P., Liu Y., Li X. (2021). Summary of Research on Geomagnetic Navigation Technology. IOP Conf. Ser. Earth Environ. Sci..

[2] Zhang X., Zhao Y. Analysis of key technologies in geomagnetic navigation. Proceedings of the International Symposium on Instrumentation and Control Technology.

[3] Li W., Wang J.L. (2014). Magnetic Sensors for Navigation Applications: An Overview. J. Navig..

[4] Khan M.A., Sun J., Li B., Przybysz A., Kosel J. (2021). Magnetic sensors—A review and recent technologies. Eng. Res. Express.

[5] Cheng Q., Sun J., Ge Y., Xue L., Mao H., Zhou L., Zhao J. (2023). Bionic Magnetic Sensor Based on the MagR/Cry4 Complex-Configured Graphene Transistor with an Integrated On-Chip Gate. ACS Sens..

[6] Cheng Q., Ge Y., Lin B., Zhou L., Mao H., Zhao J. (2024). Capacitive Bionic Magnetic Sensors Based on One-Step Biointerface Preparation. ACS Appl. Mater. Interfaces.

[7] Winklhofer M. (2009). The Physics of Geomagnetic-Field Transduction in Animals. IEEE Trans. Magn..

[8] Kirschvink J.L., Gould J.L. (1981). Biogenic Magnetite as a Basis for Magnetic-Field Detection in Animals. BioSystems.

[9] Hore P.J., Mouritsen H. (2016). The Radical-Pair Mechanism of Magnetoreception. Ann. Rev. Biophys..

[10] Zoltowski B.D., Chelliah Y., Wickramaratne A., Jarocha L., Karki N., Xu W., Mouritsen H., Hore P.J., Hibbs R.E., Green C.B. (2019). Chemical and structural analysis of a photoactive vertebrate cryptochrome from pigeon. Proc. Natl. Acad. Sci. USA.

[11] Hochstoeger T., Al Said T., Maestre D., Walter F., Vilceanu A., Pedron M., Cushion T.D., Snider W., Nimpf S., Nordmann G.C. (2020). The biophysical, molecular, and anatomical landscape of pigeon CRY4: A candidate light-based quantal magnetosensor. Sci. Adv..

[12] Li H., Liu M., Liu K., Zhang F. (2018). A Study on the Model of Detecting the Variation of Geomagnetic Intensity Based on an Adapted Motion Strategy. Sensors.

[13] Jun Z., Qiong W., Cheng C. (2019). Geomagnetic gradient bionic navigation based on the parallel approaching method. Proc. Inst. Mech. Eng. Part G-J. Aerosp. Eng..

[14] Liu M.Y., Liu K., Peng X.G., Li H. Bio-inspired navigation based on geomagnetic for the autonomous underwater vehicle. Proceedings of the OCEANS 2014.

[15] Guo J.J., Liu M.Y., Wang M.F., Zhou X.X., Yang Y. Bio-inspired Geomagnetic Navigation Algorithm Based on Segmented Search for AUV. Proceedings of the 2018 IEEE International Conference on Robotics and Biomimetics (ROBIO).

[16] Zhang Y., Liu X., Luo M., Yang C. (2021). Bio-Inspired Approach for Long-Range Underwater Navigation Using Model Predictive Control. IEEE Trans. Cybern..

[17] Zhou Y.Q., Niu Y., Liu M.Y. Bionic Geomagnetic Navigation Method for AUV Based on Differential Evolution Algorithm. Proceedings of the 2022 Ocean. Hampton Roads.

[18] Lü Y., Song T. (2013). Avian magnetoreception model realized by coupling a magnetite-based mechanism with a radical-pair-based mechanism. Chin. Phys. B.

[19] Shi H., Tang R., Wang Q., Song T. (2024). Application of bioinspired geomagnetic sensor measurements and geomagnetic map modeling based on neural networks in simulated navigation. Meas. Sci. Technol..

[20] Gill J.P., Taylor B.K. (2024). Navigation by magnetic signatures in a realistic model of Earth’s magnetic field. Bioinspiration Biomim..

[21] Hinton G., Deng L., Yu D., Dahl G.E., Kingsbury B. (2012). Deep Neural Networks for Acoustic Modeling in Speech Recognition: The Shared Views of Four Research Groups. ISPM.

[22] Krizhevsky A., Sutskever I., Hinton G. (2012). ImageNet Classification with Deep Convolutional Neural Networks. Adv. Neural Inf. Process. Syst..

[23] Chen Z., Liu K., Zhang Q., Liu Z., Chen D., Pan M., Hu J., Xu Y. (2023). Geomagnetic Vector Pattern Recognition Navigation Method Based on Probabilistic Neural Network. IEEE Trans Geosci Remote Sens.

[24] Ma X., Zhang J., Li T. (2024). Geomagnetic reference map super-resolution using convolutional neural network. Meas. Sci. Technol..

[25] Haykin S. (1994). Neural Networks: A Comprehensive Foundation.

[26] Rumelhart D.E., Hinton G.E., Williams R.J. (1986). Learning representations by back-propagating errors. Nature.

[27] Hinton G.E., Osindero S., Teh Y. (2006). A Fast Learning Algorithm for Deep Belief Nets. Neural Comput..

[28] Alken P., Erwan T., Beggan C.D., Amit H., Aubert J., Baerenzung J., Bondar T.N., Brown W., Califf S., Chambout A. (2020). International Geomagnetic Reference Field: The thirteenth generation. Earth Planets Space.

